# Antibody recognition of the glycoprotein g of viral haemorrhagic septicemia virus (VHSV) purified in large amounts from insect larvae

**DOI:** 10.1186/1756-0500-4-210

**Published:** 2011-06-21

**Authors:** Paloma Encinas, Silvia Gomez-Sebastian, Maria Carmen Nunez, Eduardo Gomez-Casado, Jose M Escribano, Amparo Estepa, Julio Coll

**Affiliations:** 1 INIA, SGIT - Dept Biotecnología Crt. Coruña Km 7 - 28040 Madrid, Spain; 2 Alternative Gene Expression S.L. (ALGENEX) Centro Empresarial Campus Montegancedo. P. Cientifico Tecnologico. UPM, 28223 Pozuelo de Alarcon Madrid, Spain; 3 IBMC - Universidad Miguel Hernández, 03202 Elche (Alicante), Spain

**Keywords:** VHSV, glycoprotein, insect larvae, ELISA, trout, antibodies, large-scale

## Abstract

**Background:**

There are currently no purification methods capable of producing the large amounts of fish rhabdoviral glycoprotein G (gpG) required for diagnosis and immunisation purposes or for studying structure and molecular mechanisms of action of this molecule (ie. pH-dependent membrane fusion). As a result of the unavailability of large amounts of the gpG from viral haemorrhagic septicaemia rhabdovirus (VHSV), one of the most dangerous viruses affecting cultured salmonid species, research interests in this field are severely hampered. Previous purification methods to obtain recombinant gpG from VHSV in *E. coli*, yeast and baculovirus grown in insect cells have not produced soluble conformations or acceptable yields. The development of large-scale purification methods for gpGs will also further research into other fish rhabdoviruses, such as infectious haematopoietic necrosis virus (IHNV), spring carp viremia virus (SVCV), hirame rhabdovirus (HIRRV) and snakehead rhabdovirus (SHRV).

**Findings:**

Here we designed a method to produce milligram amounts of soluble VHSV gpG. Only the transmembrane and carboxy terminal-deleted (amino acid 21 to 465) gpG was efficiently expressed in insect larvae. Recognition of G21-465 by ß-mercaptoethanol-dependent neutralizing monoclonal antibodies (N-MAbs) and pH-dependent recognition by sera from VHSV-hyperimmunized or VHSV-infected rainbow trout (*Oncorhynchus mykiss*) was demonstrated.

**Conclusions:**

Given that the purified G21-465 conserved some of its most important properties, this method might be suitable for the large-scale production of fish rhabdoviral gpGs for use in diagnosis, fusion and antigenicity studies.

## Background

Membrane glycoproteins (gpGs) are highly relevant in rhabdoviral infections [1]. Consequently, considerable effort has been devoted to the structural and functional analysis of these molecules [2,3]. However, large-scale purification methods for fish gpGs have not been developed to date [4]. More concretely, production and purification techniques to obtain fish rhabdoviral gpGs in milligram amounts for either immunisation or diagnosis, as well as for studying their structure/functionality (ie.: fusion) are required. In this regard, research into viral haemorrhagic septicaemia rhabdovirus (VHSV), one of the rhabdoviruses affecting aquacultured salmon and trout species [5], is severely hindered by the lack of a system with the capacity to produce sufficient amounts of the gpG. Thus, the development of new tools for detecting gpG-induced antibodies (Abs) to follow up DNA vaccinations, the estimation of anti-gpG Abs in survivors to control asymptomatic carrier fish movements, and the study of the relevance of their lineal/conformational epitopes in the protection of DNA vaccines, among others, would be facilitated by techniques able to produce milligram amounts of purified gpG. However, previous attempts to purify gpG using affinity chromatography with immobilized monoclonal Abs (MAbs)[6], concanavalin A [7] or recombinant gpG from yeast [8-10] have met with only partial success. Although these approaches gave a highly purified form of the gpG, the yield was low and the isolated protein showed a strong tendency to precipitate out of solution. Furthermore, injection of fish with recombinant rhabdoviral gpGs produced in *E. coli *[11,12], yeast [11] or baculovirus grown in insect cells [13] did not induce satisfactory immune responses. This finding was attributed mostly to the precipitable conformations of these molecules at physiological pH.

Although the gpG of fish rhabdoviruses has been obtained in small amounts in insect cell cultures and insect larvae by infection with recombinant baculoviruses [13-17], neither the large-scale production of insect-produced VHSV gpG nor its antigenic properties have been reported yet.

Here we used insect larvae to produce milligram amounts of VHSV gpG in soluble form and in a conformation as close as possible to its native structure. After several attempts, VHSV gpG using a transmembrane and carboxy terminal-deleted (amino acid 21 to 465) gpG sequence (G21-465) was the only sequence of this protein that we succeeded in expressing in large amounts. We show here that G21-465 is recognized by conformation-dependent (ß-mercaptoethanol-dependent) neutralizing monoclonal antibodies (N-MAbs) and by sera from VHSV-immunized trout in a pH-dependent manner. On the basis of our findings, we propose that given that recombinant G21-465 conserved some of its native properties and conformations, this purification procedure might serve to produce sufficient fish rhabdoviral gpGs for diagnosis, fusion and antigenicity studies.

## Findings

### Hypothesis

Recombinant VHSV gpG can be obtained in soluble milligram amounts using baculoviruses grown in insect larvae. This recombinant form binds to ß-mercaptoethanol-dependent neutralizing-monoclonal antibodies (N-MAbs) and shows pH-dependent conformational changes.

## Methods

The construction and use of plasmid pMCV1.4-G coding for the gpG (G1-507) of viral haemorrhagic septicemia (VHSV) strain VHSV-0771, isolated in France from rainbow trout (*Oncorhynchus mykiss*) [18], was previously described [19,20]. The DNA sequences corresponding to G21-507 and G21-465 without their VHSV signal sequence were amplified by PCR. The pMCV1.4-G plasmid was used for template and the 5'GCGGATCCTCAGATCACTCAACGACC (forward) and the 5'GCTCTAGAGACCGTCTGACTTCTGGAG (reverse) for G21-507 or the 5' GCTCTAGATGATGGCCAAAGACTCCAG (reverse) for G21-465 were used for primers. For directional subcloning, sense and antisense primers included *BamHI/XbaI *(underlined) restriction sites, respectively [21]. The PCR reaction was carried out by a denaturation step at 95°C for 5 min, followed by 30 cycles of amplification (94°C for 20 s, 52°C for 30 s, 72°C for 1 min) and an extension at 72°C for 10 min.

Subcloning of the amplified DNA was done in frame with the insect signal sequence derived from honey bee melittin into the multiple cloning site of the pFastMelB2 plasmid baculovirus transfer vector (derived from the pFastBac1™ of Invitrogen). The subcloned amplified DNA was flanked by 2 Tn7 transposon donor sites (Tn7 flanked genes). The pFastMelB2 plasmid also contained an ampicilin resistance gene and a poly histidine (polyHis) coding sequence of 8 amino acids added to the carboxy end (5' TCTAGACATCACCACCACCATCACTAA) of the recombinant gene protein. The recombinant pFastMelB2 constructs were transfected into DH5α *E.coli *and ampicilin-resistant clones were isolated following standard methods.

Recombinant baculoviruses were then constructed using the Bac-to-Bac^® ^baculovirus expression system (Invitrogen) and following the manufacturer's instructions. The Bac-to-Bac site-specific transposition system uses the DH10 Bac™strain of *E. Coli*, which contains a Tn7 helper plasmid to insert exogenous Tn7-flanked genes into the Tn7 target site of its 150-kb bacmid DNA. Briefly, the pFastMelB2 recombinant constructs containing the Tn7-flanked G21-507 or G21-465 sequences were first transfected into DH10 Bac™-competent *E. coli *cells. Transformed colonies were then identified by antibiotic selection and blue/white screening (since Tn7 transposition disrupts *lac*Zα). High molecular weight DNAs containing the recombinant bacmids were then prepared from selected *E. coli *clones.

Next, recombinant baculoviruses were produced in *Spodoptera frugiperda *(Sf21) insect cells (Invitrogen, Barcelona, Spain). Thus, Sf21 cells cultured in Grace's insect media (Gibco BRL) with 10% foetal bovine serum, 3% non-essential amino acids and 20 μg/ml gentamicin at 28°C [22] were co-transfected with the recombinant bacmids and wild-type AcMNPV baculovirus using lipofectamine^® ^(Gibco BRL). Seventy two hours after transfection, supernatants containing the recombinant baculoviruses were collected and amplified by re-infecting fresh Sf21 insect cells. As a negative control, the wild-type baculovirus with no insert (BacNi) was used.

*Trichoplusia ni *(cabbage looper) larvae [18-20] were bred as previously described [22,23] and used to grow the recombinant baculoviruses. Briefly, *Trichoplusia ni *eggs were placed in larvae developmental cages at 22°C, 50% humidity, under a 8 h/day-light period and fed an insect diet [24] for 10-14 days. Instar larvae were sedated for 15 min by incubation on ice and injected in the proleg with 10 μl of ~ 30 × 10^6 ^pfu/ml of the corresponding recombinant baculoviruses with the aid of a semi-automatic injector. Larvae injected with BacNi baculovirus were used as a negative control. Inoculated larvae were maintained at 28°C, and 72 h later, when they became swollen, pale and lethargic, they were frozen immediately at -70°C until processing.

**Figure 1:**
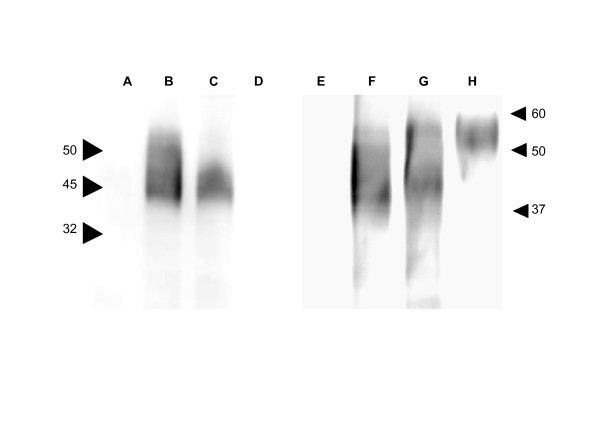
**Recognition of extract proteins from *Trichoplusia ni *insect larvae infected with recombinant baculoviruses BacNi (lanes A and E), G21-465 (lanes B and F), G21-507 (lanes C and G) and concentrated VHSV (lanes D and H) with anti polyHis (lanes A, B, C, D) or anti-gpG (lanes E, F, G, H) MAbs**. Larvae were collected 72 hours after infection with the recombinant baculoviruses. Larvae were homogenized in guanidinium-free buffer and their soluble proteins were extracted. Thirty μg of protein extracts was loaded and electrophoresed in the same 12% polyacrylamide gel (PAGE) and transferred to a nitrocellulose membrane. The membrane was then cut and reacted with anti-His MAb (lanes A, B, C and D) or with anti-gpG MAb mix (lanes E, F, G and H) and peroxidase labelled anti-mouse Ig and then detected by chemiluminiscence. Some of the molecular weight markers on KDa are shown by the arrow numbers to the left and to the right.

Using immunoblotting to detect specific gpG epitopes in the G21-507 and G21-465 sequences derived from insect, 30 μg of insect protein extract per lane was loaded in 12% SDS-polyacrylamide gels, separated, and then transferred to nitrocellulose membranes (Schleicher & Schuell). These membranes were first treated overnight with a blocking solution of phosphate buffer saline-0.05% Tween 20 (PBS-T) with 4% skin milk. They were then cut in 2 parts contining 4 lanes each and each part was incubated for 1 h at room temperature with an anti-poly His MAb (Sigma) or with 50-fold diluted anti-G MAb mixture (see below). After washing with PBS-T, lanes were incubated with 200-fold diluted anti-mouse horseradish peroxidase-conjugated IgGs (Sigma). Finally, the bands were visualized by chemiluminiscence with an ECL kit (Amersham) (Figure 1).

The anti-VHSV gpG-specific monoclonal antibodies (MAbs) C10 [25,26], I16 (INRA, unpublished), 3F1A12 and IPIH3 (unpublished) were obtained from the French INRA (Dr. M. Bremont) and the Denmark Centre of Aarhus (Dr. N. Lorenzen), respectively. At least 500 ml of hybridoma culture supernatants was purified by affinity chromatography on protein A to 1 mg/ml of IgG. An equimolar mixture of purified C10, I16 and 3F1A12 was used to detect VHSV gpG in the immunoblots.

**Figure 2:**
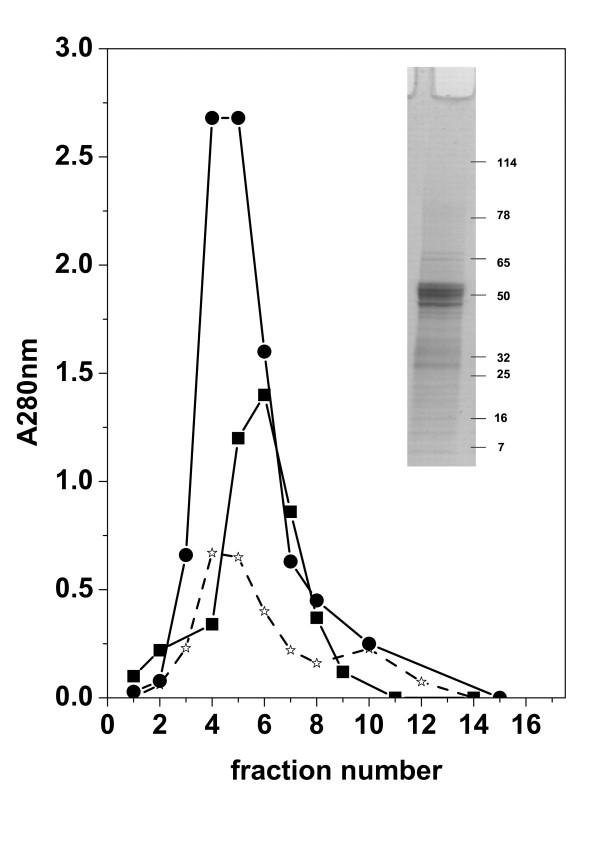
**Ni-affinity chromatography fractions of protein extracts from *Trichoplusia ni *insect larvae infected with recombinant baculoviruses, and PAGE of the pooled fractions of G21-465 (insert)**. Fifty recombinant baculovirus-infected larvae (~10 g) were homogenized in 6 M guanidinimun chloride, 1 M sodium chloride in 40 mM phosphate buffer at pH 7.8 containing 25 mM imidazole. They were then disrupted by sonication and centrifuged until a clear lysate was obtained. A 3-ml Probond (Invitrogen) bed column was used to retain the polyhistidine-tagged recombinant proteins. Bound proteins were eluted using the same buffer with 250 mM imidazole. ●, protein extract from larvae infected with the G21-465 recombinant baculovirus. ■, protein extract from larvae infected with the G21-507 recombinant baculovirus. *, protein extract from larvae infected with the BacNi baculovirus. Fractions with an absorbance > 0.3 at 280 nm were pooled and electrophoresed in a 4 to 20% polyacrylamide gel. Results of G21-465 stained with Coomassie-blue are shown in the insert.

To obtain purified recombinant polyhistidine-tagged proteins by Ni2+ affinity chromatography, 50 recombinant baculovirus-infected larvae (~15 g) were homogenized in 6 M guanidinimun chloride, 1 M sodium chloride in 40 mM phosphate buffer at pH 7.8 containing 25 mM imidazole and a protease inhibitor cocktail (Complete, Roche). The extracts were then homogenized at 10 W with a needle Virtis sonicator until a clear lysate was obtained. After removing the pellet by centrifugation at 10000 *g *for 10 min, the supernatants were filtered through Miracloth paper to remove the lipidic fraction (Calbiochem). A 3-ml bed Probond (Invitrogen) column was used to retain the polyhistidine-containing recombinant proteins. Bound proteins were then eluted using the same buffer with 250 mM imidazole. To maximize yields, fractions with absorbances > 0.3 at 280 nm (Figure 2: fractions 3, 4, 5, 6 and 7 for G21-507 and 3, 4, 5, 6, 7 and 8 for G21.465) were pooled. To prevent their precipitation, these fractions were immediately adjusted to pH 4, chromatographed through a Sephadex G-100 column in 20 mM sodium acetate pH 4 and extensively dialyzed against 20 mM sodium acetate pH 4. Protein concentrations were determined using the bicinchoninic acid (BCA) method [27] and purity was confirmed by densitometry of Coomassie-blue stained protein bands separated on 10-20% polyacrylamide gradient gels (PAGE, BioRad). The purified recombinant proteins were kept frozen until use.

To estimate the binding of MAbs and trout anti-VHSV gpG Abs to purified G21-465, each well in 96-well MaxiSorb polystyrene plates (Nunc) was coated with 2 μg of the native or reduced ß-mercaptoethanol purified recombinant G21-465 in 100 μl of carbonate buffer pH 9.6 overnight at 4°C [10]. To reduce the disulphide bonds of G21-465, 200 μg of protein in 200 μL of H_2_O was incubated with 120 mM ß-mercaptoethanol for 2 min at 100°C. Forty μl of 500 mM iodoacetamide were then added in 0.1 M sodium bicarbonate and the mixture was incubated for 30 min at 37°C. The final mixture was dialyzed against 20 mM sodium acetate pH 4. The coated wells were then blocked for 1 h with 100 μl/well of dilution buffer (0.5% bovine serum albumin, 0.1% Tween 20, 0.01% merthiolate, 0.005% phenol red in phosphate buffered saline pH 6.7 or 7.7) and washed with 0.05% Tween 20 immediately before performing the ELISA. The wells were incubated with 100 μl/well of MAbs or trout sera diluted to 2.5 μg/well or 200-fold in dilution buffer, respectively, for 60 min at room temperature. After washing, they were incubated for 30 min with anti-trout immunoglobulin MAb 1G7 [28] and anti-mouse horseradish peroxidase-conjugated IgGs (Sigma) and developed with o-phenylenediamine, as described before [29].

VHSV 07.71 was grown in epithelial papullosum cyprini (EPC) cells and concentrated from infected EPC supernatants by using 7% polyethylene glycol (PEG) 6000 in 2.3% NaCl, pH 7.8, as described previously [7,8].

Sera from VHSV-immunized rainbow trout (*Oncorynchus mykiss*) were used to study the pH-dependent recognition of the purified G21-465 by fish Abs. Thus, hyperimmunized sera from 3 rainbow trout (hyperimmunized rainbow trout sera, hTS) intraperitoneally injected with 30 μg of purified VHSV 07.71 were obtained as described before [9], by following the experimental protocols approved by the corresponding Ethics Committee of the *Departamento de Biotecnologia *(INIA). Sera from VHSV 07.71-infected trout survivors with their corresponding neutralization titers (50% plaque neutralization tests, PNT) were gifts from Dr. Castric (AFSSA, France). Pool1 corresponded to the 3 hTS, pool 2 corresponded to 3 sera from VHSV-infected trout with non-detectable (< 40) neutralization titers, while pool 3 corresponded to sera from 4 VHSV-infected trout with neutralization titers > 5120.

## Results

The corresponding gpG gene sequence between amino acids 21-507 (complete gpG) was first cloned into the pFastMelB2 plasmid. The recombinant G21-507 gene under the control of the polyhedrin promoter, using an insect signal sequence derived from honey bee melitin and adding a carboxy terminal poly histidine (polyHis) tag for affinity purification, was included in recombinant baculoviruses. However, preliminary results showed that G21-507 was expressed in low yields when the corresponding recombinant baculovirus was injected into insect larvae, it precipitated out of solution when purified, and it was not stable after freezing. The alternative G21-465 sequence, devoid of their transmembrane and carboxy-terminal domains, was then designed, cloned and expressed in baculovirus.

To identify recombinant gpG protein expression, specificity was confirmed by immunoblotting using commercial anti-polyHis MAb and an anti-G MAbs mixture (C10, I16 and 3F1A12) (Figure 1). In most preparation batches (n = 5), electrophoresed and immunoblotted protein extracts from insect larvae injected with G21-465 recombinant baculovirus showed 3-4 unresolved individual bands between 44 to 56 KDa which were similar with both MAbs (Figure 1 lanes B and F, respectively). Those injected with G21-507 showed 2-3 unresolved individual bands between 44 to 46 KDa with anti-polyHis MAb or 44 to 56 KDa with anti-G MAbs mixture (Figure 1 lanes C and G, respectively). Native G1-507 obtained from concentrated VHSV, showed either no bands with anti-polyHis MAb and 2-3 unresolved individual bands between 50 to 58 KDa when reacted with the anti-G MAbs mixture (Figure 1 lanes D and H, respectively). No major immune-reactive bands in the 32-60 KDa region were detected in the extracts from insect larvae injected with the BacNi baculovirus (Figure 1 lanes A and E). The corresponding theoretical molecular weights of the amino acid backbone sequences corresponding to the recombinant G21-465 and G21-507 without any modification (glycosilation) and including the polyHis tails were 51.499 and 55.868 Da (54.814 Da of the viral G21-507 plus 1054 Da of the polyHys tail), respectively. The small differences and/or the variety of apparent molecular weights displayed in the immunoblotting bands could be explained by distinct glycosylation patterns. Alternatively, minor immunoreactive bands with lower molecular weights could also be derived from proteolytic cleavages of the main protein bands.

Figure 2 shows the absorbance profiles obtained by Ni2+ affinity chromatography purification of protein extracts from G21-465, G21-507 and BacNi baculovirus-injected insect larvae in 6 M guanidinimun chloride and 1 M sodium chloride (denaturing buffer). The amount of purified G21-465 was 4-5-fold higher than the amount of G21-507. We estimated the amount of insect larvae proteins in the BacNi control to be 6- to 7- and 4- to-5-fold lower than G21-465 and G21-507 proteins, respectively. The G21-465 purified protein migrated as 4 main bands in gradient polyacrylamide PAGE with apparent molecular weights of 57, 55.5, 50.5 and 47 KDa (insert of Figure 2). Further purification by sephadex chromatography reduced the amounts of the smaller molecular weight bands (not shown). Upon frozen storage, G21-507 precipitated out of solution and could not be redisolved using non-denaturing buffers. In contrast, we were able to freeze and thaw the G21-465 sequence several times without it precipitating out of solution. Given the instability shown by G21-507, we selected G21-465 to continue with characterization studies. Thus, further preparative experiments after affinity and sephadex chromatography yielded 15 mg of purified G21-465 (an estimated mean of 0.3 mg per larvae, n = 50) with > 85% of purity. Parallel purifications using non-denaturing buffers (buffers in the absence of guanidinimun and sodium chlorides) for the affinity chromatography resulted in very low yields of G21-507 and G21-465.

Although the G21-465 expression product was in a disulphide-intact conformation, it could also be partially denatured after affinity purification in the presence of high concentrations of guanidinium. Therefore, we applied ELISA to study the recognition of G21-465 by conformation-dependent/-independent MAbs at both physiological (7.7) and fusion (6.7) pHs. Because the wild-type gpG has 8 disulphide bonds [25], in order to estimate the disulphide conformation dependence of the MAb binding, we also estimated binding in the presence and absence of ß-mercaptoethanol. Given that earlier observations showed a higher immunoblotting reactivity between sera from VHSV-immunized trout and recombinant fragments of gpG (not published), we also examined the binding of sera from VHSV-immunized trout at a range of pHs.

**Figure 3:**
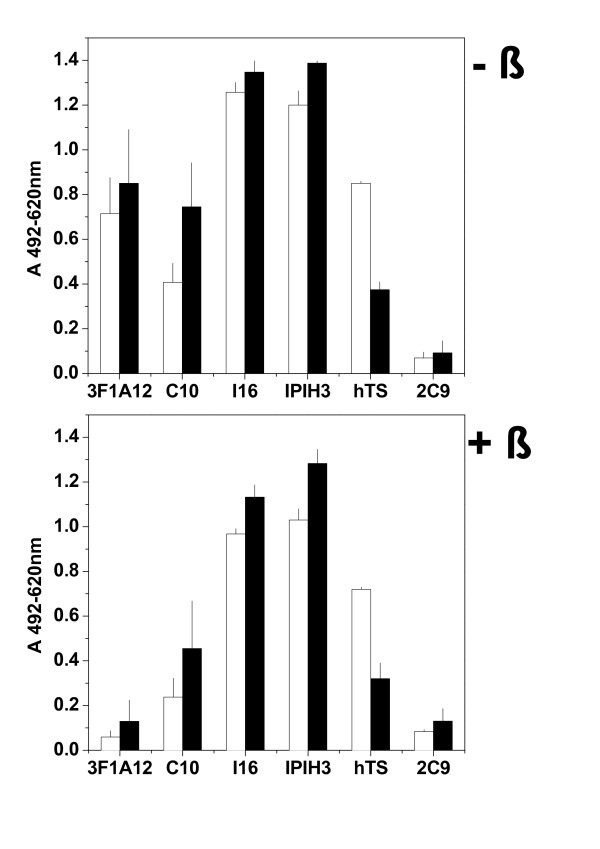
**Recognition of solid-phase G21-465 by anti-gpG MAbs and VHSV-hyperimmunized trout sera (hTS)**. Plates were coated with 1 μg per well of insect-derived G21-465 untreated (- ß) or treated with ß-mercaptoethanol (+ ß), as indicated in the text. Hyperimmunized trout sera (hTS) were obtained by intraperitoneal injections of purified VHSV and diluted 2000-fold. The MAb 2C9 was an anti-VHSV nucleocapsid N-MAb used as a negative control [29]. MAbs were used at 2.5 μg per well. Open bars, ELISA performed with dilution buffer adjusted to pH 6.7. Black bars, ELISA performed with dilution buffer adjusted to pH 7.7. Means and standard deviations from 4 experiments are shown.

Solid-phase immobilized G21-465 was recognized by anti-gpG MAbs 3F1A12, C10, I16 and IPIH3 and by sera from trout hyperimmunized with VHSV (Figure 3). Reduction of the disulphide bonds of G21-465 decreased their recognition by 3F1A12 and C10 8- and 2-fold, respectively, while the binding of MAbs I16 and IP1H3 remained unchanged. The 3F1A12 MAb epitopes mapped to position 253 (N. Lorenzen, personal communication), a position flanked by 2 close cystein residues forming a disulphide bridge [25], while the C10 MAb epitopes mapped simultaneously to 2 separated amino acid positions in the gpG sequence: 140 and 433 [26]. Therefore, it was expected that 3F1A12 and C10 binding would be affected by ß-mercaptoethanol treatment. In contrast, I16 and IPIH3, which were insensitive to this treatment, mapped at lineal epitopes located at amino acid positions 139-153 and 399-413, respectively [9]. These results show that some of gpG conformation-dependent functional activity was preserved in the G21-465 recombinant protein. This finding is most probably attributable to slow refolding during sephadex chromatography and dialysis, since G21-465 precipitated when these steps were omitted or performed at physiological pH.

Similar MAb binding profiles were obtained when the ELISAs were done using dilution buffer at pH 6.7 (fusion) and 7.7 (physiological)(Figure 3), except for C10, which showed lower binding at pH 6.7. In contrast, the binding of sera from hyperimmunized rainbow trout (hTS) to G21-465 by ELISA (Figure 3) or of several immunized trout sera by immunoblot blot (Table 1) at pH 6.7 were 2-3-fold higher than binding at pH 7.7. Results were similar with pooled hyperimmunized trout sera (trout intraperitoneally injected with purified VHSV) and with pooled trout sera surviving VHSV infection with undetectable neutralization titers (< 40) or with high neutralization titers (> 5120). Given that the conformational changes at pH 6.7 affect many epitopes of gpG [8,20], these results were also expected when using polyclonal Abs such as those from immunized trout sera. Previous immunoblotting to detect anti-G Abs in VHSV-survivor trout sera has been performed at alkaline pHs [30,31], thus their reduced reactivity under these conditions might explain the failures to detect anti-G Abs by immunoblotting in some sera from VHSV-immunized trout [30,31].

**Table 1:** pH-dependence of the recognition of G21-465 by sera from hyperimmunized (pool 1) and VHSV-infected (pool 2 and pool 3) rainbow trout as shown by immunoblotting

Trout sera	Immunization with VHSV-07.71	Number of sera	Neutralization titer	pH 6.7/pH 7.7, ratio
Pool 1	Hyperimmunized	3	800	2.85 ± 1.36 (n = 3)
Pool 2	Surviving infection	3	< 40	3.45 ± 0.23 (n = 2)
Pool 3	Surviving infection	4	> 5120	3.12 ± 0.58 (n = 2)

G21-465 was electrophoresed in 5-20% polyacrylamide gels (gradient PAGE), transferred to nitrocellulose and derived strips were used for reaction with each of the pooled trout sera at pH 6.7 and 7.7 as described, except that diaminobenzidine (DAB) was used for staining the reaction. Pool 1 included 3 sera from trout hyperimmunized by injection of purified VHSV with neutralization titers of 800 (dilution of the sera causing a 50% decrease in the *in vitro *VHSV titer). Pool 2 included 3 sera from trout surviving infection with VHSV with neutralization titers < 40 (gift of Dr.Castric). Pool 3 included 4 sera from trout surviving infection with VHSV with neutralization titers > 5120 (gift from Dr. Castric). The intensity of the stained bands were estimated by densitometry (Image J 1.38x, http://rsb.info.nih.gov/ij) and ratios were then calculated by the formula: intensity of the stained bands at 6.7/intensity of the stained bands at pH 7.7. Means and standard deviations were calculated from at least 2 experiments (n).

The production of G21-465 in insect larvae and their down-stream purification were straightforward procedures and did not require disruption of the native disulphide structure of the protein. In contrast, for the isolation in soluble form of a yeast-derived recombinant gpG (G4) [11], unfolding by high temperature in the presence of elevated concentrations of ß-mercaptoethanol were required. Previous purification of recombinant frg11 (amino acid 56-110) designed from the fusion-related domain of VHSV gpG [20,32,33] showed its potential application for ELISA [10], a possibility which was recently confirmed by further studies [34]. From the practical point of view, the production of recombinant gpG in insect larvae was as reproducible as recombinant frg11 in *E. coli *[10], with the added advantage that most of the gpG epitopes, including the disulphide-dependent epitopes, were conserved only in the insect system. We propose that the conservation of most of the gpG disulphide structure will allow new applications for this recombinant protein, among these use for the detection of disulphide-dependent conformational epitopes. The presence of a samll histidine tag in the G21-465 molecule is not expected to interfere with ELISA, as this has not been reported for other recombinant proteins obtained in this system [21-23].

To be used for solid-phase in ELISA, purified VHSV [7], purified gpG from VHSV [7] or recombinant unfolded yeast G4 protein [8-10], met with difficulties, mainly as a result of their low yields and tendency to precipitate. Given that the G21-465 sequence obtained by using the purification procedure described here it is expected to maintain all its disulphide-dependent [25] epitopes, both in the pre-fusion and post-fusion conformations [2,3] and it can be obtained in large amounts in soluble form, it might be suitable for antigenicity studies and for the development of detection methods for fish anti-gpG Abs.

## Competing interests

The authors declare that they have no competing interests.

## Authors' contributions

PE and EG purified the G21-465 and performed the ELISAs, respectively. SG and MC carried out the baculovirus constructs and infections. JME participated and coordinated the baculovirus work. AE and JC coordinated the whole study and wrote the manuscript. All the authors read and commented on the final manuscript.
